# Editorial: miRNAs and Neurological Diseases

**DOI:** 10.3389/fneur.2021.662373

**Published:** 2021-04-20

**Authors:** Hsiuying Wang, Y. H. Taguchi, Xianshuang Liu

**Affiliations:** ^1^Institute of Statistics, National Yang Ming Chiao Tung University, Hsinchu, Taiwan; ^2^Department of Physics, Chuo University, Hachioji, Japan; ^3^Henry Ford Hospital, Detroit, MI, United States

**Keywords:** microRNA, neurological disease, biomarker, target genes, therapeutics

microRNAs (miRNAs) are short non-coding RNAs that play an important role in many biological processes including cellular proliferation, differentiation, maturation, the maintenance of immune homeostasis, and normal cellular function. miRNAs have been linked to many human diseases including cancer. They are involved in the pathogenesis of neurodegenerative disorders such as Alzheimer's disease, amyotrophic lateral sclerosis, and Parkinson's disease (1, 2). In addition to neurodegenerative disorders, miRNAs are related to many other neurological diseases such as spinal muscular atrophy, Prader–Willi syndrome, Niemann–Pick disease, neurofibromatosis, narcolepsy, Friedreich's ataxia, and ataxia-telangiectasia (3). miRNAs are promising drug targets for neurological diseases (4, 5). The circulating miRNA biomarkers of prognosis have been identified for many neurological disorders and can be used as prognosis biomarkers of neurological diseases, meaning miRNA-related pathogenesis may help new drug development. Drug resistance has been a critical issue for many diseases. miRNA therapy could provide new hope for patients with therapeutic or drug resistance issues. Moreover, miRNA can be used to explore the association between neurological diseases and tumors and the association between diseases and vaccination (6, 7). They also are involved in the mechanisms of immunoregulation and inflammation in autoimmune diseases. Nevertheless, the role of miRNAs in the mechanisms of these neurological diseases is not yet characterized.

In recent years, there have been many noteworthy studies looking at the role of miRNAs in neurological disorders. These have examined miRNA functions *in vitro* and *in vivo* model systems, and have identified miRNA biomarkers, miRNA regulatory networks, miRNA pathways, and looked at miRNA therapeutics. These valuable studies shed light on the effective diagnosis and treatment of neurological disorders and have revealed the importance of studying miRNAs and their role in neurological diseases.

This Research Topic aims to examine the role of miRNA in neurological diseases. The special issue brings together five interesting papers, including three Reviews, a Systematic Review, and a Perspective article, discussing the role of miRNAs in multiple sclerosis, Alzheimer's disease, diabetic neuropathy, and Clathrin-Mediated Endocytosis.

The Systematic Review by Zhou et al. performed a meta-analysis, aiming to assess the overall diagnostic accuracy of circulating miRNAs for multiple sclerosis, an immune-mediated chronic inflammatory demyelinating disease of the central nervous system. They searched publications from PubMed, Web of Science, EMBASE, the Cochrane Library, Chinese National Knowledge Infrastructure databases, and related papers up to July 20, 2019, to collect 600 patients with multiple sclerosis and 389 controls. These results suggest that miRNAs had reference value for multiple sclerosis diagnosis.

Alzheimer's disease is the most common form of dementia among older people and accounts for around 60% of all dementia cases. miRNAs are easily detected in brain and immunological cell types. Kou et al. reviewed the dysfunctional regulation of a series of miRNAs that were associated with the critical roles in the pathogenesis of Alzheimer's disease including the dysfunctional regulation of miRNAs associated with the deposition of Aβ, intracellular aggregation of hyperphosphorylated Tau protein, the loss of synapses, neuroinflammation, autophagic dysfunction, and aging. The perspective article written by Lukiw reviewed the current understanding of miRNA-146a, attributing to neurological diseases including Alzheimer's disease and prion disease. This study discusses the critical role of signaling along the NF-kB–miRNA-146a axis in brain cell fate. Clathrin-Mediated Endocytosis is a vital process for cell life and development. Gerasymchuk et al. discussed the role of miRNA regulating endocytic genes in neurodegeneration and cancer/invasion. They concluded that some endocytic-associated miRNAs with strictly proven roles in cancer and neurodegeneration are worth paying more attention to as possible therapeutic targets. Diabetic neuropathy is the most prevalent chronic complication of diabetes mellitus. miRNA-based treatment of diabetic neuropathy has shown evidence of therapeutic potential. Fan et al. reviewed the latest research progress on the roles of miRNAs as biomarkers and as potential clinical therapeutic targets in diabetic neuropathy. Moreover, the promise of exosomal miRNAs as therapeutics was discussed and recommendations for future research on miRNA-based medicines were provided.

In summary, these five papers provide valuable research or review works in understanding the relationship between miRNAs and neurological disorders. We hope this special issue can contribute to a better understanding of the potential of miRNA therapeutics for neurological diseases.

## Author Contributions

All authors listed have made a substantial, direct and intellectual contribution to the work, and approved it for publication.

## Conflict of Interest

The authors declare that the research was conducted in the absence of any commercial or financial relationships that could be construed as a potential conflict of interest.
